# Supplementary material for: Activation of VGLL4 Suppresses Cardiomyocyte Maturational Hypertrophic Growth
Source: Cells. 2024 Aug 13;13(16):1342. doi: 10.3390/cells13161342 (PMC11352427; doi:10.3390/cells13161342)
Supplement: Supplementary file 1 [file cells-13-01342-s001.zip › cells-2932544-6_Supplemental information_Final_ZL.pdf]

Activation of VGLL4 suppresses cardiomyocyte maturational hypertrophic growth

Aaron Farley[a], Yunan Gao[a,b], Yan Sun[a], Sylvia Zohrabian[c], William T. Pu[c], and Zhiqiang Lin[a],*

Supplemental information

Extended material and methods

Plasmids.

TEAD1-Flagbio and VGLL4-GFP plasmids were previously published [1]; 8xGTIIC-luciferase was a gift from Stefano Piccolo (Addgene plasmid # 34615). For the pCMA-HA-hCTSB plasmid, the coding sequence of human mature CTSB was first amplified with the following primers: 5'-aacGAATTCCTGCCTGCAAGCTTCGATGCACGGGAA-3', 5'-aagCTCGAGCTGATCGGTGCGTGGTATTCCAGCC-3', and the PCR products were digested with EcoRI and XhoI. After gel purification, the hCTSB sequence was cloned in frame into the pCMA-HA vector, with HA tag coding sequence positioned in the 5 prime side of hCTSB.

AAV preparation

pAAV.cTnT.VGLL4^{K225R} and pAAV.cTnT.Luciferase were previously described [1]. AAV9 was packaged in 293T cells with AAV9:Rep-Cap and pHelper (pAd deltaF6, Penn Vector Core) and purified and concentrated by gradient centrifugation. AAV9 titer was determined by quantitative PCR.

Gene and protein expression analysis

Total RNA was isolated with Trizol. For quantitative reverse transcription polymerase chain reaction (qRT-PCR), RNA was reversely transcribed (Superscript III) and specific transcripts were measured using SYBR Green chemistry (Lifesct., M0016-25) and normalized to glyceraldehyde 3-phosphate dehydrogenase (*Gapdh*). Primer sequences were provided in the Supplementary Table 1. Antibodies used for immunofluorescence staining and western blots were listed in Supplementary Table 2.

NRVMs immunofluorescence staining and cell size measurement.

NRVMs were fixed with 4% paraformaldehyde (PFA) for 20 minutes at room temperature. After washing with PBS and permeabilized with PBST (PBS+ 0.1% Triton x100) solution, cells were incubated with cardiac troponin I type 3 (TNNI3) antibody (Suppl. Table 2) for immunofluorescence staining. Images were acquired with a Keyence BZX-700 microscope, and the cell sizes were measured with Image J.

Luciferase reporter assay

HEK 293T cells were cultured in 24 well plates for luciferase assay. 50 ng/well indicated plasmids and 10 ng pRLTK internal control vector (Promega) were transfected with 1µl 1mg/ml

Polyethylenimine (PEI) (Polysciences, 25414-2.) Luciferase activity was measured 24 hours after transfection using the Dual-Luciferase reporter assay system (Promega).

TEAD1 Co-immunoprecipitation (Co-IP)

Cell protein extracts for CoIP were prepared in lysis buffer (20 mM Tris-HCl [pH 8], 137 mM NaCl, 10% glycerol, 1% Triton X-100, and 2 mM EDTA) supplemented with protease inhibitor cocktail (Roche). The protein solution was diluted with 1 volume of immunoprecipitation buffer (lysis buffer without glycerol), to which 15 µL Flag-antibody conjugated Dynabeads (Thermo Fisher) were added to pull down FLAG-tagged TEAD1 and its interaction proteins. After three washes, the immunoprecipitated proteins were eluted with 1x SDS loading buffer and applied for western blot analysis.

Myofibril measurements

NRVMs were cultured on Laminin (10µg/ml) coated glass cover slips. After hormone treatment, cells were fixed with 4% PFA for 2 hours before being used for TNNI3 immunofluorescence staining. Stained NRVMs were imaged with a Nikon AXR Confocal system equipped with a 60x objective lens.

Reference:

[1] Lin, Z., Guo, H., Cao, Y., Zohrabian, S., Zhou, P., Ma, Q., VanDusen, N., Guo, Y., Zhang, J., Stevens, S.M., Liang, F., Quan, Q., van Gorp, P.R., Li, A., Dos Remedios, C., He, A., Bezzerides, V.J. and Pu, W.T. (2016) Acetylation of VGLL4 Regulates Hippo-YAP Signaling and Postnatal Cardiac Growth. *Dev Cell* **39**, 466-479.

Supplementary Table S1. qRT-PCR primer list

Species	Gene name	Forward	Reverse
Rat	*Pik3ca*	AAAATGACAAGGAACAGCTCCG	GCAGTACATCTGGGCCACTTC
Rat	*Pik3cb*	GGGGAAGCGTGGGGCACATG	AGGTCAGAGAGCGCCTCCCG
Rat	*Tead1*	AGAGACGGAGTATGCGAGGT	GCTGTGCTCCATGCTCACTA
Rat	*Yap*	TGTCAGACCGTCAGAGCGGGAA	GCTGAGGCCACTGTCTGTGCTC
Rat	*Myh7*	GAGGAGAGGGCGGACATT	ACTCTTCATTCAGGCCCTTG
Rat	*Myh6*	TCAAACTGGAGCTGGATGAC	GTATTCATTGGCCTGGTCCT
Rat	*Gapdh*	ATCACCATCTTCCAGGAGCGA	AGCCTTCTCCATGGTGGTGAA
Rat	*Lpl*	AGGTGGACATCGGGGAATTG	CCCTGGCACAGAAGATGACC
Rat	*Cd36*	AGATGCAGCCTCCTTTCCAC	AGAACAAATCTGTACACGGGGA
Rat	*Pnpla2*	AACGCCACTCACATCTACGG	TACCAGGTTGAAGGAGGGGT
Rat	*SLC27A*	CTTCTGCGAGAACCCGTGAG	CTACCCACGTACACACCGAA
Rat	*Fabp4*	CTTTGTGGGGACCTGGAAACT	ATGACGACCAAGTCCCCTTC
Rat	*Fabp3*	ACCGACATCGACCTCCTTTC-	ATTGTGGTCGGCTTGGTCAT
Rat	*Cpt1a*	CCGAGAAGGGAGGACAGAGA	GTACAGGTGCTGGTGCTTCT
Rat	*Ctsb*	GTGTCTGACAGCGAGAAGGA	TGCTACCAGCCAGTAGGGTA
Mouse	*Cd36*	TCCTCTGACATTTGCAGGTCTATC	AAAGGCATTGGCTGGAAGAA
Mouse	*Lpl*	GGGAGTTTGGCTCCAGAGTTT	TGTGTCTTCAGGGGTCCTTAG
Mouse	*SLC27A*	GCTCAGAACTTCCCAGTCCA	CCACCCACGTACACACAGAA
Mouse	*Fabp4*	GCAGACGACAGGAAGGTGAA	TCCTTTGGCTCATGCCCTTT
Mouse	*Fabp3*	agtcactggtgacgctggacg	aggcagcatggtgctgagctg
Mouse	*Pnpla2*	CAACGCCACTCACATCTACGG	GGACACCTCAATAATGTTGGCAC
Mouse	*Cpt1a*	GGACTCCGCTCGCTCATT	GAGATCGATGCCATCAGGGG
Mouse	*Gapdh*	CAGGTTGTCTCCTGCGACTT	GGCCTCTCTTGCTCAGTGTC

Supplementary Table S2. Primary Antibody list

Primary Antibody	Host	Vendor (Cat #)	Usage (dilution)
TNNI3 Ab	Goat	Abcam (ab169826)	IF (1:250)
β-actin Ab	Mouse	Santa Cruz (sc-47778)	Western blot (1:1000)
Phospho Akt Ab	Rabbit	CST (#9271)	Western blot (1:1000)
Akt Ab	Rabbit	CST (#9272)	Western blot (1:1000)
GFP Ab	Goat	Rockland (600-101-215)	Western blot (1:1000)
GAPDH Ab	Mouse	Fitzgerald (10R-G109a)	Western blot (1:10,000)

Flag Ab	Mouse	Sigma (F1804)	Western blot (1:1000)
ATGL Ab	Rabbit	CST (#2138)	Western blot (1:1000)
CTSB	Rabbit	Thermofisher (PAS-14255)	Western blot (1:1000)

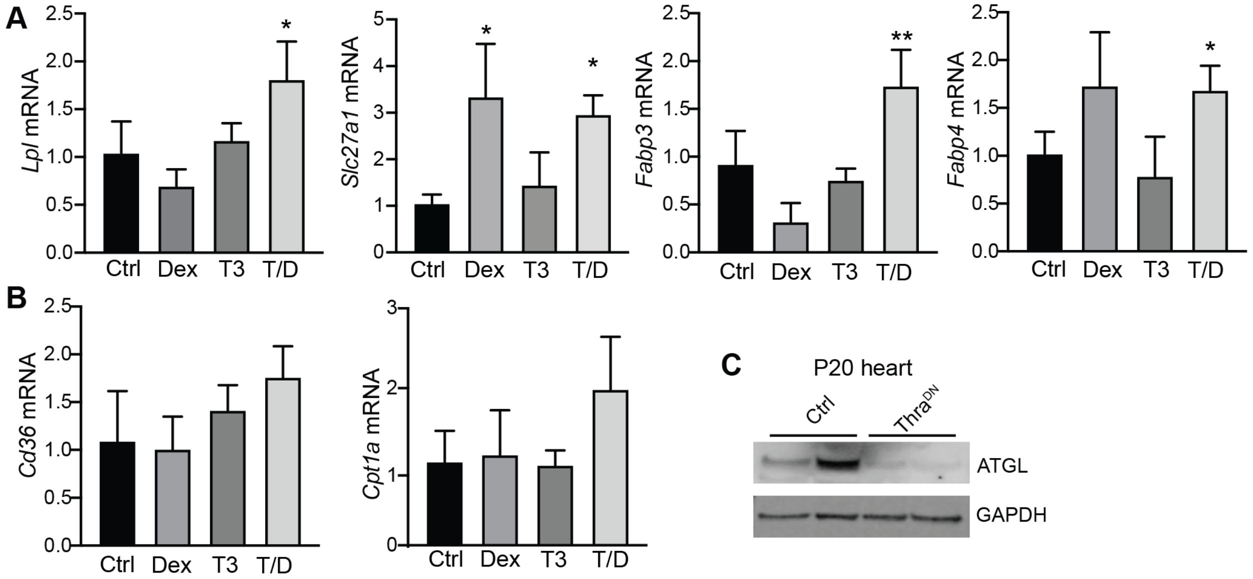

Suppl. Figure S1. Related to Figure 2. **A-B.** qRT-PCR measurement of gene expression. 2 days after indicated treatmet, NRVMs were collected for gene expression analysis. One Way ANOVA followed by Tukey post hoc multiple comparison analysis: *, $p<0.05$. N=4. **C**. Western blot of ATGL. Protein extracted from the hearts of postnatal day 20 mice were analyzed. GAPDH was used as loading control.

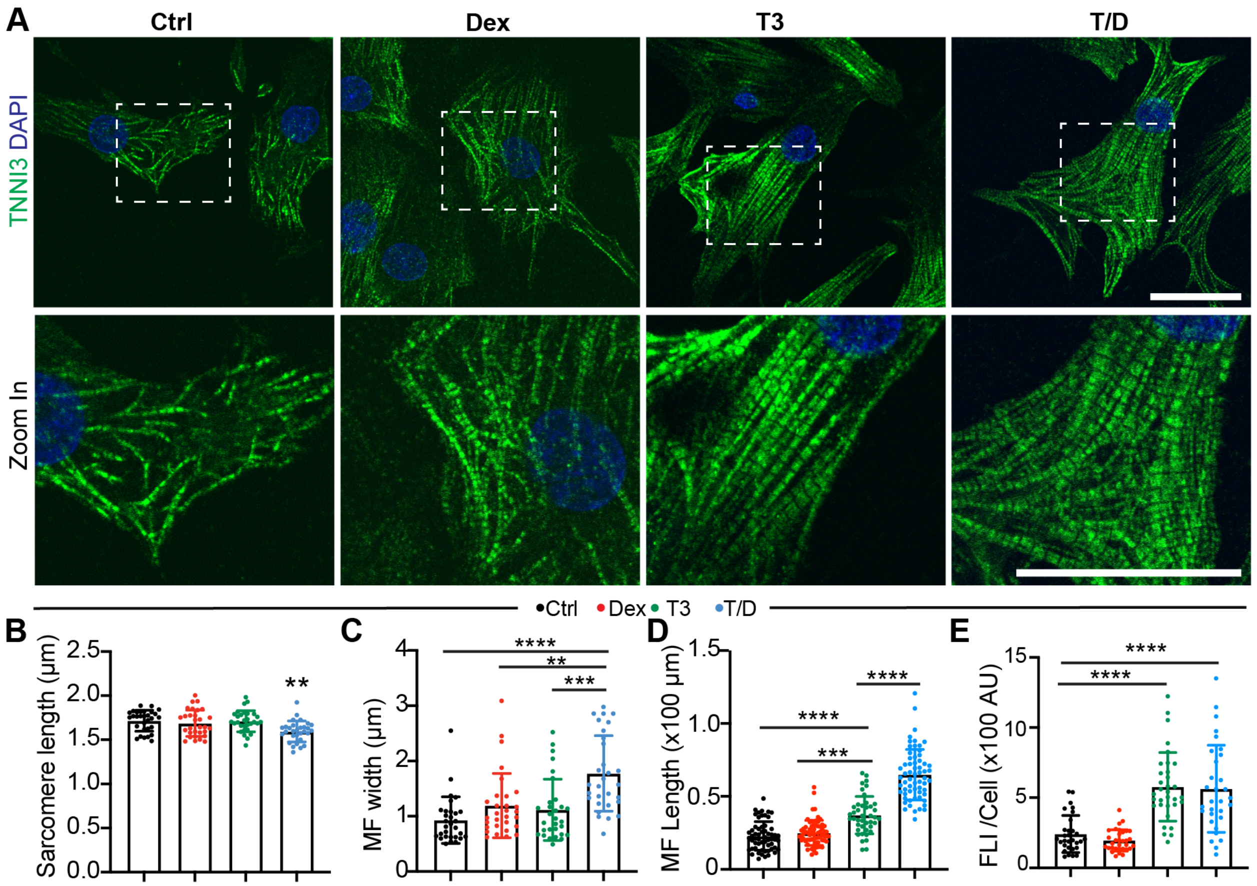

Suppl. Figure S2. Related to Figure 2. T3 and Dex promotes the formation of organized myofibrils. **A**. Immunofluorescence images of NRVMs. Scale bar = 25 μm.

B–E. Quantification of sarcomere length (B), Myofibril (MF) thickness (C), Length (D) and TNNI3 fluorescence intensity (FLI) (E). B, C, and D, each data point indicates the average value of three myofibrils/cell. C and D, statistical analysis was performed with Kruskal–Wallis ranks test followed by Dunn's multiple comparison test. **, $p<0.01$; ***, $p<0.001$; ****, $p<0.0001$. B and C, for each group, n=30 cells. D, *n*=60 cells for each group. E, AU, arbitrary unit. For each group, *n*=30.

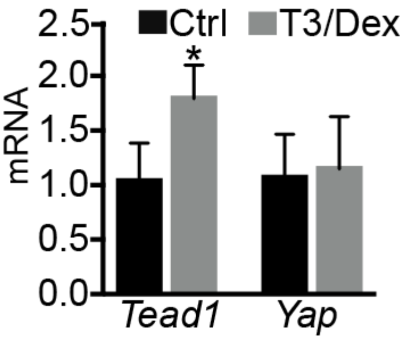

Suppl. Figure S3. Related to Figure 3. qRT-PCR measurement of *Tead1* and *Yap* gene expression. 2 days after indicated treatment, NRVMs were collected for gene expression analysis. Student's t-test *, $p<0.05$. N=4.

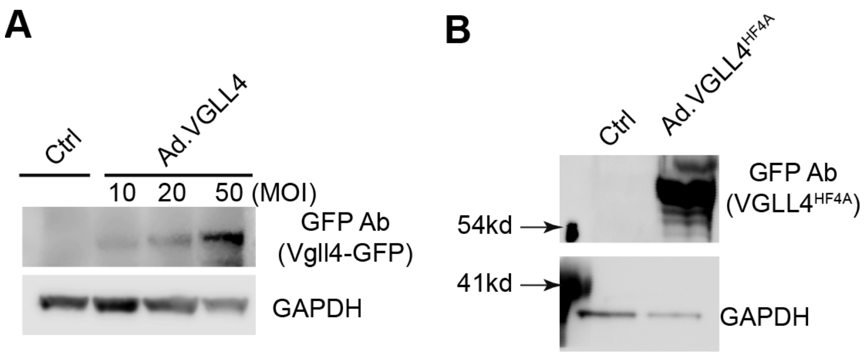

Suppl. Figure S4. Related to Figure 4 and Figure 6.

A. Western blot of VGLL4-GFP. **B.** Western blot of VGLL4^{HF4A}**.** A and B, protein extracted from control and Adenovirus infected H9C2 cells was used to detect the expression of VGLL4-GFP (A) and VGLL4^{HF4A} (B). A, MOI: multiplicity of infection GAPDH was used as loading control.

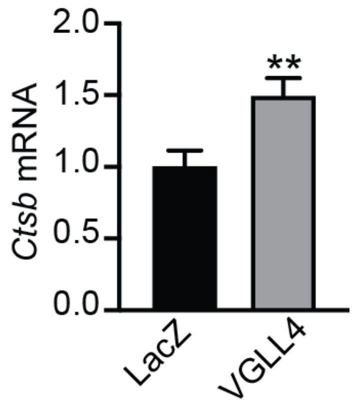

Suppl. Figure S5. Related to Figure 8. qRT-PCR measurement of *Ctsb*. 2 days after indicated treatmet, NRVMs were collected for RNA isolation. Student's t-test *, $p<0.05$. N=4.
