# Supplementary material for: Activation of VGLL4 Suppresses Cardiomyocyte Maturational Hypertrophic Growth
Source: Cells. 2024 Aug 13;13(16):1342. doi: 10.3390/cells13161342 (PMC11352427; doi:10.3390/cells13161342)

Original blots

Figure 2I

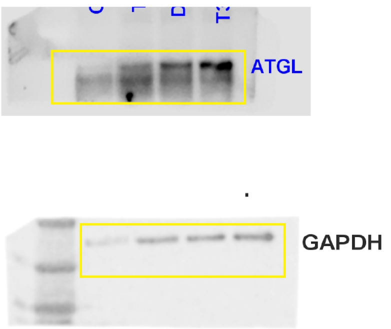

Figure 4C

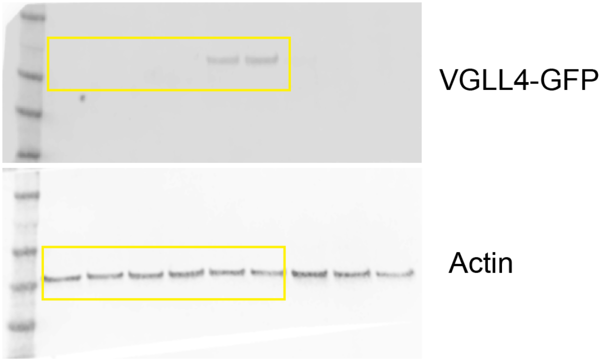

Figure 6B

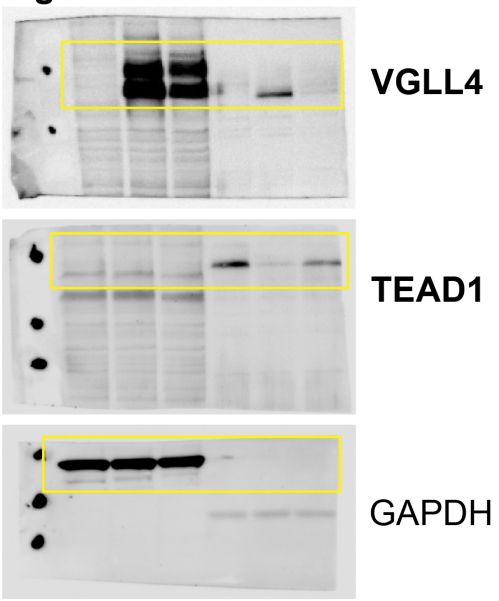

Figure 7B

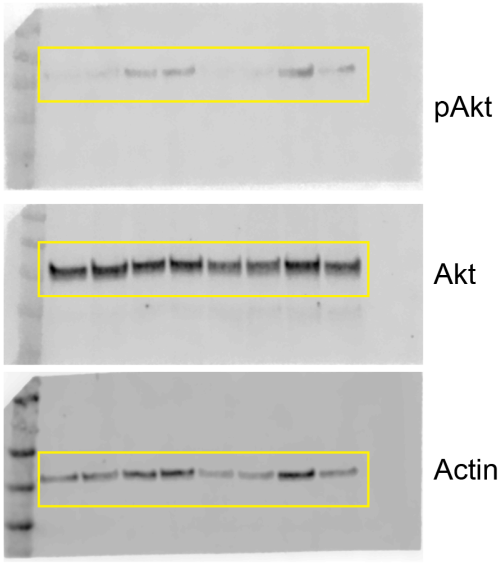

Figure 8A

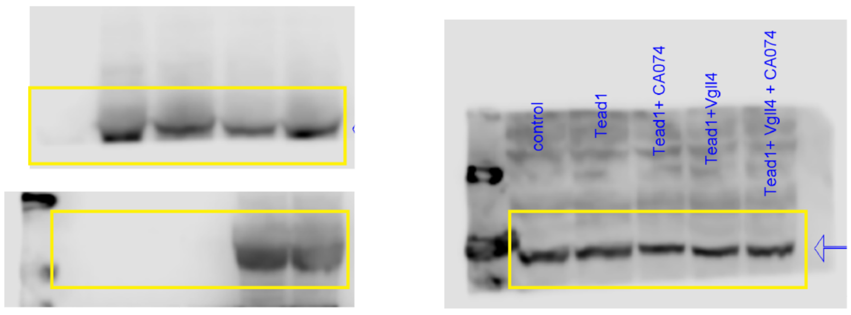

Figure 8C

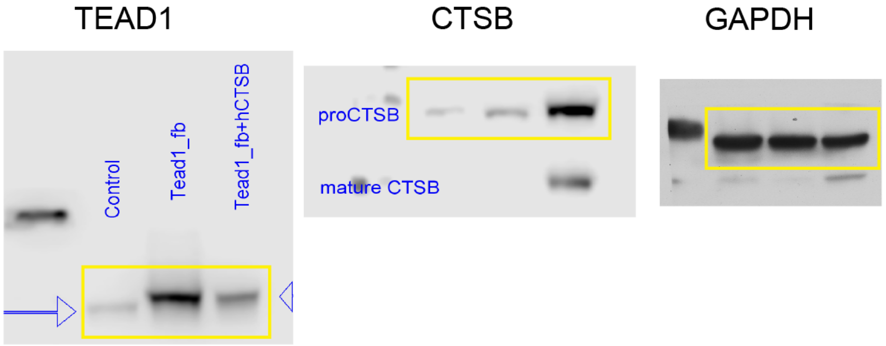

Figure 8D

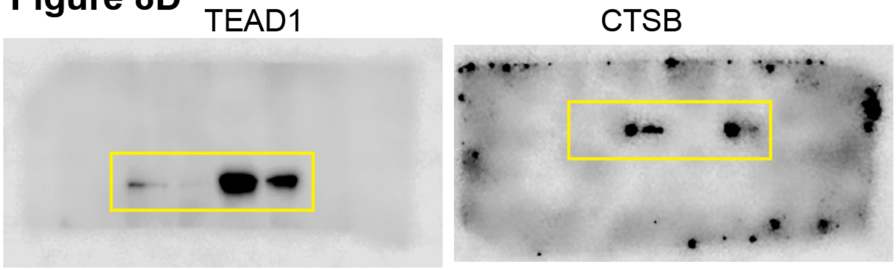

Figure 8E

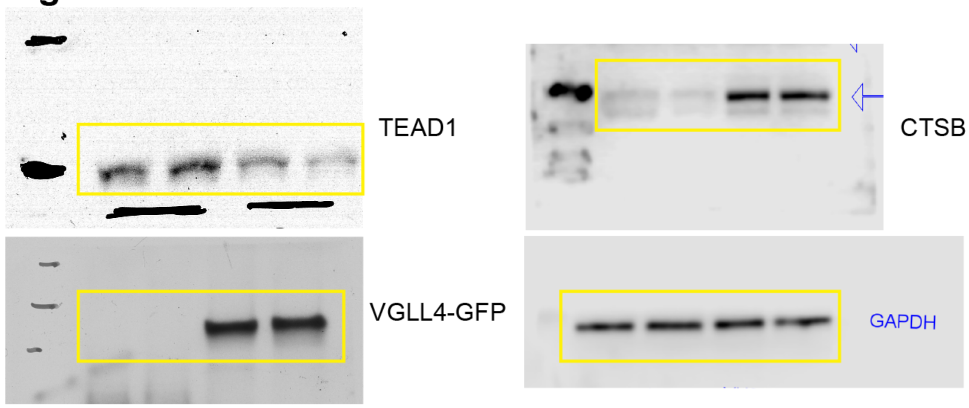

Suppl. Figure 1C

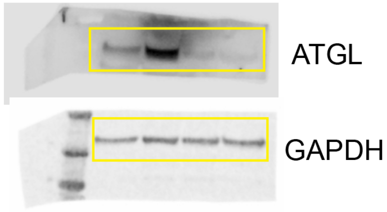

Supplemental Figure 4A

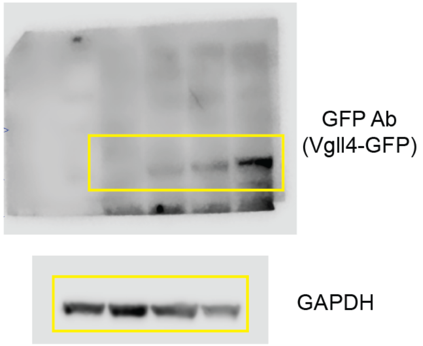

Supplemental Figure 4B

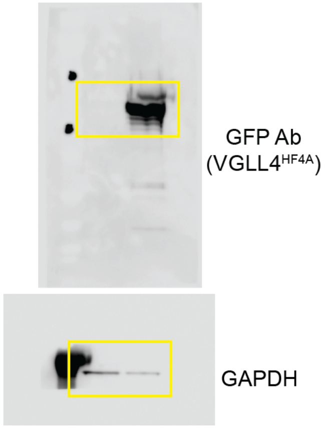

Supplement: Supplementary file 1 [file cells-13-01342-s001.zip › Original blots_All blots.pdf]
